# Dynamic cycling with a unique Hsp90/Hsp70-dependent chaperone machinery and GAPDH is needed for heme insertion and activation of neuronal NO synthase

**DOI:** 10.1016/j.jbc.2022.102856

**Published:** 2022-12-31

**Authors:** Yoshihiro Morishima, Miranda Lau, William B. Pratt, Yoichi Osawa

**Affiliations:** Department of Pharmacology, University of Michigan Medical School, Ann Arbor, Michigan, USA

**Keywords:** nitric oxide synthase, molecular chaperones, Hsp90, Hsp70, GAPDH, heme, ARS, ATP-regenerating system, BSA, bovine serum albumin, CaM, calmodulin, DSP, dithiobis(succinimidylpropionate), GR, glucocorticoid receptor, Hsp, heat shock protein, IgG, immunoglobulin G, iNOS, inducible NOS, NO, nitric oxide, NOS, nitric oxide synthase, nNOS, neuronal NO synthase, sGC, soluble guanylate cyclase

## Abstract

Heat shock protein 90 (Hsp90) is known to mediate heme insertion and activation of heme-deficient neuronal nitric oxide (NO) synthase (apo-nNOS) in cells by a highly dynamic interaction that has been extremely difficult to study mechanistically with the use of subcellular systems. In that the heme content of many critical hemeproteins is regulated by Hsp90 and the heme chaperone GAPDH, the development of an *in vitro* system for the study of this chaperone-mediated heme regulation would be extremely useful. Here, we show that use of an antibody-immobilized apo-nNOS led not only to successful assembly of chaperone complexes but the ability to show a clear dependence on Hsp90 and GAPDH for heme-mediated activation of apo-nNOS. The kinetics of binding for Hsp70 and Hsp90, the ATP and K^+^ dependence, and the absolute requirement for Hsp70 in assembly of Hsp90•apo-nNOS heterocomplexes all point to a similar chaperone machinery to the well-established canonical machine regulating steroid hormone receptors. However, unlike steroid receptors, the use of a purified protein system containing Hsp90, Hsp70, Hsp40, Hop, and p23 is unable to activate apo-nNOS. Thus, heme insertion requires a unique Hsp90–chaperone complex. With this newly developed *in vitro* system, which recapitulates the cellular process requiring GAPDH as well as Hsp90, further mechanistic studies are now possible to better understand the components of the Hsp90-based chaperone system as well as how this heterocomplex works with GAPDH to regulate nNOS and possibly other hemeproteins.

The function and turnover of a wide variety of signaling proteins are regulated by heat shock protein (Hsp) 90^3^ (reviewed in Ref. (1)). These Hsp90 client proteins constantly undergo cycles of Hsp90 heterocomplex assembly and disassembly in the cytoplasm and nucleus. Hsp90 heterocomplex assembly is directed by an Hsp90/Hsp70-based chaperone machinery that regulates the client signaling proteins by modulating ligand-binding clefts (reviewed in Ref. (2)). Cycling with the chaperone machinery also regulates client protein turnover. Hsp90 and Hsp70 have opposing effects in that Hsp90 stabilizes client proteins, and, when Hsp90 is inhibited, degradation occurs *via* the ubiquitin–proteasome pathway (3), which is initiated by Hsp70-dependent E3 ubiquitin ligases, such as CHIP (C terminus of Hsc70-interacting protein) (4) and parkin (5).

The nitric oxide synthase (NOS) enzymes, including endothelial NOS, neuronal NOS (nNOS), and inducible NOS (iNOS), are signaling proteins whose activity is enhanced by Hsp90 (6, 7, 8, 9, 10, 11, 12, 13). These enzymes are cytochrome P450-like hemeproteins that catalyze the conversion of l-arginine to nitric oxide (NO) and citrulline by a process that requires NADPH and molecular oxygen (14). NOS enzymes are bidomain in structure with an oxygenase domain, which contains the binding sites for heme, substrate, and tetrahydrobiopterin, and a reductase domain, which contains the binding sites for FMN, FAD, and NADPH. NOS enzymes are highly regulated, requiring homodimerization and binding of Ca^2+^–calmodulin (CaM) for activity. Hsp90 binds to the oxygenase domain of NOS enzymes (15, 16), and studies with purified proteins show that CaM and Hsp90 increase binding of each other to both endothelial NOS and nNOS (10, 11, 13, 17). The binding of CaM to NOS enhances electron flux from flavin bound to the reductase domain to heme bound within the cleft (18).

For the past 20 years, our laboratory has studied the action of Hsp90 and Hsp70 on nNOS (reviewed in Refs. (19, 20)). Treatment of cells with Hsp90 inhibitors decreases nNOS catalytic activity, increases nNOS turnover, and inhibits activation of heme-deficient nNOS (apo-nNOS) by exogenous heme (7). nNOS degradation is *via* the ubiquitin–proteasome pathway (5, 7, 21, 22). Reaction of certain mechanism-based inactivators in the heme/substrate-binding cleft causes opening of the cleft to yield a more unfolded state of the protein (2, 20), triggering nNOS ubiquitination and proteasomal degradation (21, 23). Hsp90 and Hsp70 have opposing effects on nNOS ubiquitination, with Hsp70 stimulating and Hsp90 inhibiting (16, 24). We have spent considerable effort in establishing the factors in cytosol that promote heme insertion into apo-nNOS. Two activation systems exist—one discovered because of an artifact of the oxidation of nNOS during purification and involves a thiol–disulfide exchange that is independent of Hsp90, and the second is an Hsp90-dependent facilitation of heme insertion uncovered by studies with intact cells.

Heme insertion into purified apo-nNOS can be activated with high concentrations of dithiothreitol and heme in the presence of superoxide dismutase and catalase to protect the heme (25). This occurs as an artifact of enzyme purification in which a disulfide bond(s) forms in a fraction of apo-nNOS during purification that stabilizes the chaperone-mediated open state of the heme/substrate-binding cleft. The resulting disulfide bond(s) prevents heme entry, but activation to the heme-bound holoenzyme dimer can be brought about by DTT or by factors present in Sf9 cytosol in a manner independent of subsequent cycling with Hsp90 (26). Both Hsp70 and thioredoxin were shown to be components of the activation system, suggesting a model in which Hsp70 binding to apo-nNOS stabilizes the open state of the heme/substrate-binding cleft to facilitate thioredoxin access to the active site cysteine that coordinates with heme iron, permitting heme binding and dimerization to the active form (27). This calls to mind an early study by Zgoda *et al.* (28) showing that hemin-mediated restoration of CYP2B1 is carried out by a chaperone-dependent and glutathione-mediated thiol–disulfide exchange.

It has proven to be difficult to define a mechanistic basis with use of subcellular systems for the observation that treatment of cells with an Hsp90 inhibitor, such as geldanamycin or radicicol, inhibits activation of apo-nNOS by heme. This inhibition of heme activation was noted in Sf9 cells, which have very low levels of endogenous heme. Addition of exogenous heme to the culture medium causes an increase in nNOS activity in infected cells (7). The ferrous carbonyl complex of nNOS is not formed when Hsp90 is inhibited, indicating that functional heme insertion into nNOS is not occurring in the inhibitor-treated Sf9 cells (29). In cellular studies, the Stuehr laboratory has shown that Hsp90 binds to apo-iNOS to facilitate heme insertion (30). Also in cellular studies, the Stuehr laboratory has shown that Hsp90 binds to the β1 subunit of soluble guanylate cyclase (apo-sGCβ1) to promote heme insertion into apo-sGCβ1, which concurrently associates with a sGCα1 partner subunit to form the mature sGC heterodimer (31, 32). The Stuehr laboratory has also shown that Hsp90 is required for heme insertion into indoleamine dioxygenase as well as globins to form mature hemoglobin and myoglobin, which suggests a role for Hsp90 in heme insertion into heme proteins in general (33, 34). In both cases of iNOS and guanylate cyclase, Hsp90 was recovered with the apo-enzyme, and very little was found with the mature heme-bound holoenzyme dimer (30, 31, 32). Similarly, Hsp90 is not recovered with mature hemoglobin (34). This has led to a model in which heme binding in the heme/substrate-binding cleft of the NOS enzymes or guanylate cyclase and heme binding to globin causes a conformational change that is unfavorable for cycling with Hsp90.

This is similar to the model developed for the glucocorticoid receptor (GR) where Hsp90 forms a quite stable complex with the unliganded receptor, and binding of steroid causes a conformational change that permits only very dynamic cycling with Hsp90, such that GR•Hsp90 heterocomplexes are not recovered from steroid-treated cells (reviewed in Ref. (2)). Compared with the unliganded GR, apo–nNOS undergoes much more dynamic cycling with Hsp90 such that very few apo-nNOS•Hsp90 heterocomplexes are recovered from mammalian cytosols when apo-nNOS is immunoadsorbed. Because this dynamic cycling with the NOS enzymes has not been demonstrated in subcellular systems that permit mechanistic analysis, we do not know if the stable Hsp90 heterocomplex assembly worked out for the steroid receptors (1, 35) and the dynamic cycling of Hsp90 with nNOS involve the same or similar Hsp90/Hsp70-dependent chaperone machineries. For example, Hsp70 is required for cycling of Hsp90 with steroid receptors (1), but we do not know if it is required for dynamic cycling of Hsp90 with nNOS. In cellular studies, both Hsp90 and the heme donor, GAPDH, have been shown to be needed for insertion of heme into iNOS, indoleamine dioxygenase, guanylate cyclase, hemoglobin, and myoglobin (33, 36, 37, 38, 39). In our prior studies with subcellular systems and in cells, heme-bovine serum albumin (BSA) was sufficient to activate apo-nNOS (25, 26, 27, 29), thus the role of GAPDH as a heme chaperone for nNOS was unclear. The goal of this work is to answer some of these mechanistic questions on Hsp90–chaperone assembly on nNOS, using three different subcellular assembly systems, as well as address the role of GAPDH as heme donor.

In the current work, we have assembled for the first time Hsp90 heterocomplexes on immobilized immunopellets of apo-nNOS with reticulocyte lysates, Sf9 insect cytosol, or purified chaperone proteins, as was done in experiments on steroid receptor–Hsp90 heterocomplex assembly (35). We show that dynamic cycling with apo-nNOS is highly similar to that for stable cycling found for steroid receptors, with Hsp70 cycling leading to Hsp90 binding in an ATP- and K^+^-dependent process. The assembled apo-nNOS•Hsp90•Hsp70 heterocomplex was shown to insert heme and activate apo-nNOS with GAPDH as the heme donor but not heme-BSA. Moreover, we have shown that heme-BSA could only activate apo-nNOS when GAPDH was present. Thus, for the first time, we have an *in vitro* system that recapitulates the cellular process, and we are now poised for further mechanistic studies to delineate how the Hsp90-based chaperone machinery and GAPDH regulate heme entry and activation of nNOS.

## Results

### Reconstitution of nNOS heterocomplexes with rabbit Hsp90 and Hsp70

**Figure 1:**
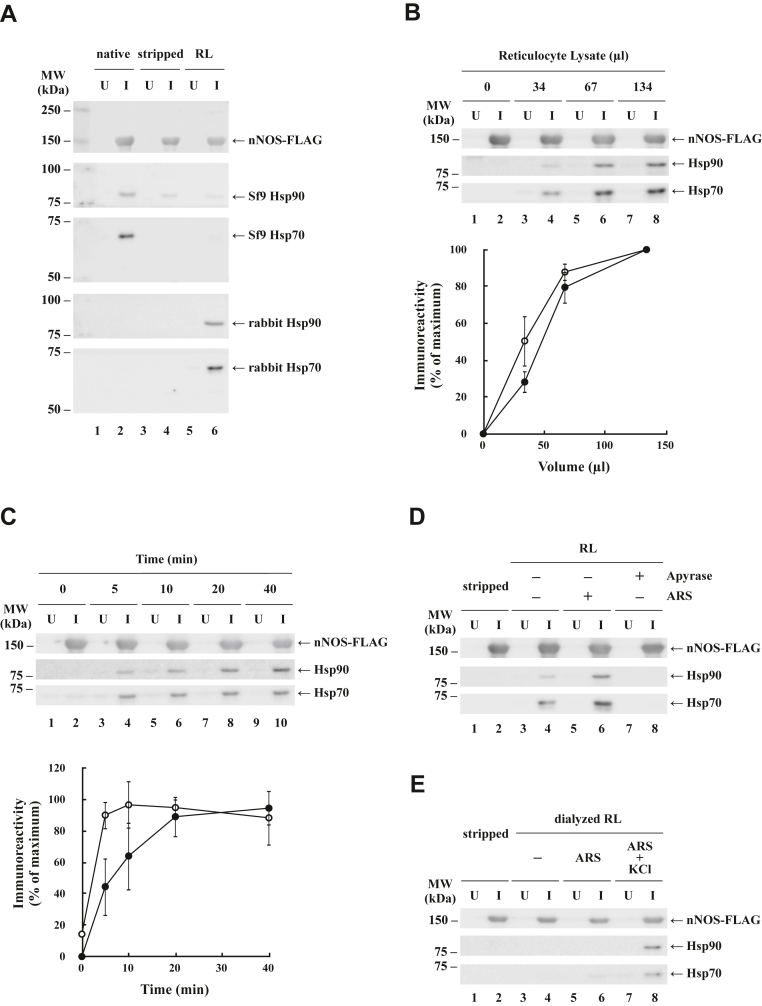
**Assembly of apo**•**nNOS•Hsp90–Hsp70 heterocomplexes with reticulocyte lysate (RL).***A*, native and reconstituted heterocomplexes. Apo-nNOS-FLAG was immunoadsorbed from uninfected (U) or baculovirus-infected (I) Sf9 cell cytosols to yield *native* heterocomplexes with Sf9 Hsp90 and Hsp70. Immunopellets were then *stripped* of insect chaperones, and stripped apo-nNOS immunopellets were incubated for 20 min at 30 °C with rabbit RL in the presence of an ATP-regenerating system (ARS). nNOS-FLAG and associated chaperones were eluted with FLAG peptide, separated by SDS-PAGE, and immunoblotted for nNOS, Sf9 Hsp90, Sf9 Hsp70, rabbit Hsp90, and rabbit Hsp70. *B*, reconstitution is dependent upon the amount of RL. Stripped apo-nNOS-FLAG immunopellets were incubated for 20 min at 30 °C with various volumes of RL. After washing, the immunopellets were treated as above, and immunoblots of Hsp90 (●) and Hsp70 (○) were quantitated by LI-COR Image Studio. In the graph, the amounts of Hsp90 and Hsp70 are expressed relative to the 134 μl value at 100% and represent the mean ± SD for three experiments. *C*, time dependence of Hsp70 and Hsp90 binding. Stripped apo-nNOS-FLAG immunopellets were incubated for the indicated times with 67 μl of RL at 30 °C, and nNOS-bound Hsp90 and Hsp70 were quantitated as above. In the graph, the relative amount of Hsp90 (●) or Hsp70 (○) was expressed as a percent of the maximum value achieved and represent the mean ± SD for three experiments. *D*, assembly of apo-nNOS complexes with Hsp90 and Hsp70 is ATP dependent. Stripped immunopellets were incubated with RL, RL supplemented with an ARS, or RL preincubated for 30 min at 30 °C with 0.2 unit/100 μl of apyrase. nNOS-FLAG was released with FLAG peptide, and nNOS, Hsp90, and Hsp70 were immunoblotted. *E*, assembly of complexes is K^+^ dependent. Stripped apo-nNOS-FLAG immunopellets were reconstituted with buffer (*stripped*; *lanes* 1 and 2), dialyzed RL (*lanes* 3 and 4), dialyzed lysate plus an ARS (*lanes* 5 and 6), or dialyzed lysate plus the ARS and 100 mM KCl (*lanes* 7 and 8). Hsp, heat shock protein; nNOS, neuronal nitric oxide synthase.

In previous experiments where purified apo-nNOS in solution was mixed with reticulocyte lysate, we could not obtain apo-nNOS•Hsp90 heterocomplexes. In the experiments shown in Figure 1, immunoadsorbed apo-nNOS-FLAG stripped of Hsp90 was incubated with rabbit reticulocyte lysate to form nNOS-FLAG•Hsp90•Hsp70 heterocomplexes. Figure 1*A* shows native apo-nNOS complexes with Sf9 Hsp90 and Hsp70, the immunopellet stripped of insect chaperones, and stripped apo-nNOS immunopellets incubated with rabbit reticulocyte lysate to reconstitute heterocomplexes containing rabbit chaperones. Over an incubation time of 20 min at 30 °C, 60 to 80 μl of reticulocyte lysate is sufficient for heterocomplex assembly (Fig. 1*B*), similar to GR•Hsp90 heterocomplex assembly with reticulocyte lysate. Figure 1*C* shows the time course for binding of both Hsp90 and Hsp70 to apo-nNOS-FLAG immunopellets. As Smith (40) reported for progesterone receptor heterocomplex assembly, Hsp70 peaks rapidly, whereas Hsp90 binding is slower. Assembly of GR heterocomplexes with Hsp90 and Hsp70 is both ATP dependent and K^+^ dependent (41), with the latter reflecting the K^+^ dependency of the ATPase activity of Hsp70 (42). As shown in Figure 1, *D* and *E*, assembly of apo-nNOS heterocomplexes with Hsp90 and Hsp70 by reticulocyte lysate is also ATP and K^+^ dependent, respectively.

Figure 1 presents several similarities between dynamic assembly of apo-nNOS•Hsp90 heterocomplexes and assembly of more stable GR•Hsp90 heterocomplexes by reticulocyte lysate. However, although the reticulocyte lysate system allowed a number of mechanistic studies of chaperone cycling on the ligand-binding activity of the GR, these are not permitted with apo-nNOS because of the very high levels of heme in the lysate. To determine the effect of chaperone cycling on heme-dependent activation of nNOS catalytic activity, we assembled apo-nNOS•Hsp90 complexes with Sf9 cytosol.

### Reconstitution of nNOS heterocomplexes with Sf9 Hsp90 and Hsp70

**Figure 2:**
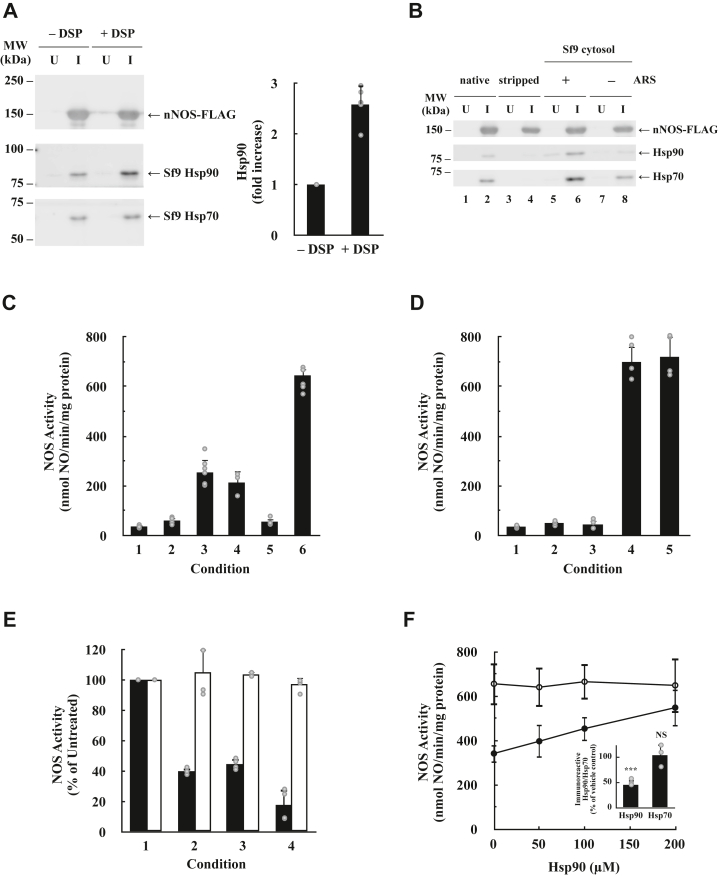
**Assembly of apo•nNOS**•**Hsp90–Hsp70 heterocomplexes with Sf9 cytosol.***A*, capture of dynamic cycling apo-nNOS-FLAG•Hsp90 complexes by treating Sf9 cells with a cell-permeable crosslinker. Sf9 cells infected for 72 h with baculovirus containing complementary DNA encoding nNOS-FLAG were harvested, washed, and treated for 30 min at room temperature in suspension with 1.5 mM dithiobis(succinimidylpropionate) (DSP) or vehicle (6% v/v dimethyl sulfoxide [DMSO]). After the crosslinking reaction was quenched with 20 mM Tris, cytosols were prepared and immunoadsorbed with anti-FLAG. nNOS-FLAG and associated Hsp90 and Hsp70 were eluted with FLAG peptide and assayed by SDS-PAGE and immunoblotting. To produce the bar graph, the Hsp90 bands from three experiments were scanned, and the amount of apo-nNOS-FLAG-bound Hsp90 from crosslinked cells was expressed as a fold increase over noncrosslinked cells set at 1. Mean ± SD for five experiments. *B*, Hsp90 binding to apo-nNOS is ATP dependent. Apo-nNOS-FLAG immunoadsorbed from uninfected (U) or baculovirus-infected (I) Sf9 cytosol (*native*) was stripped of associated chaperones and incubated for 20 min at 30 ºC with Sf9 cytosol in the presence or the absence of an ATP-regenerating system (ARS). Apo-nNOS-FLAG and associated chaperones were released with FLAG peptide and resolved by SDS-PAGE and immunoblotting. *C*, apo-nNOS chaperone complex from the Sf9 reconstitution system yields heme-dependent activation of nNOS catalytic activity. Purified apo-nNOS-FLAG was immunoadsorbed with anti-FLAG and incubated with Sf9 cytosol. Unbound proteins were washed off, and the pellets were incubated for 90 min at room temperature with the heme-containing nNOS activation system, released with FLAG peptide, and nNOS activity was measured by the oxyhemoglobin method. Immunoadsorbed purified apo-nNOS-FLAG (*lane* 1), incubated with buffer alone (*lane* 2), incubated with Sf9 cytosol with ARS (*lane* 3), incubated with Sf9 cytosol without ARS (*lane* 4), and incubated with Sf9 cytosol and ARS but without heme in the activating system (*lane* 5). The activity of immunoadsorbed and purified holo-nNOS-FLAG eluted with FLAG peptide was also assayed (*lane* 6). The nNOS activities represent the mean ± SD for five experiments. *D*, incubation of apo-nNOS-FLAG with Sf9 cytosol and the heme-dependent activating system combined in a single incubation mix yields complete enzyme activation. Immunoadsorbed purified apo-nNOS-FLAG (*lane* 1) was incubated with Sf9 cytosol alone (*lane* 2), the heme-dependent activating mix alone (*lane* 3), or with heme-activating mix and cytosol together (*lane* 4). *Lane 5*, holo-nNOS-FLAG control. FLAG peptide released material was assayed for nNOS activity. The nNOS activities represent the mean ± SD for four experiments. *E*, inhibition of Hsp90 (with radicicol) or Hsp70 (with pifithrin-μ) or both inhibits heme-mediated activation of apo-nNOS-FLAG. Apo-nNOS-FLAG was immunoadsorbed from Sf9 cytosol, washed, and incubated with Sf9 cytosol and the heme-dependent activating mix minus heme at 20 °C for 15 min in the presence of buffer (*lane* 1), 100 μM radicicol (*lane* 2), 100 μM pifithrin-μ (*lane* 3), or both inhibitors (*lane* 4). Heme/bovine serum albumin (BSA) was then added, the incubation was continued for 45 min at 20 °C, the nNOS-FLAG was released with FLAG peptide, and enzyme activity was assayed. Immunoadsorbed purified holo-nNOS-FLAG was submitted to the same procedure. *Solid bars*, apo-nNOS-FLAG; *open bars*, holo-nNOS-FLAG. The nNOS activities represent the mean ± SD for at least three experiments. *F*, purified Hsp90 reverses the inhibitory action of radicicol on heme-mediated activation of nNOS by Sf9 cytosol. The experiment was as in *E* with the use of apo-nNOS-FLAG, except that 75 μM radicicol was used, and purified Hsp90 was added in the amounts indicated. *Solid circles*, radicicol; *open circles*, vehicle. The nNOS activities represent the mean ± SD for three experiments. *Inset*, radicicol decreases the binding of Hsp90 to nNOS when nNOS•Hsp90•Hsp70 heterocomplexes are assembled with Sf9 cytosol. Immunoadsorbed and stripped apo-nNOS-FLAG was reconstituted with Sf9 cytosol in the presence of 100 μM radicicol. The amounts of Hsp90 and Hsp70 were determined by immunoblotting and are expressed as a percentage of the values with vehicle. The values represent the mean ± SD for three experiments. NS, not significant; *∗∗∗*, difference significant at *p* < 0.001. Hsp, heat shock protein; nNOS, neuronal nitric oxide synthase.

Because of their very low levels of endogenous heme, Sf9 cells infected with nNOS-FLAG baculovirus produce primarily apo-nNOS. Probably because of the high concentration of the apo-enzyme, heterocomplexes of apo-nNOS-FLAG with insect Hsp90 are readily detectible in Sf9 cytosol, despite the dynamic nature of the cycling of Hsp90 with apo-nNOS (Fig. 1*A*). This contrasts with nNOS expressed in human embryonic kidney 293 cells where we have to use an intracellular crosslinker to reliably demonstrate nNOS•Hsp90 heterocomplexes (16). The dynamic nature of cycling of Hsp90 with apo-nNOS-FLAG is illustrated in Figure 2*A*, where Sf9 cells expressing nNOS-FLAG were treated for 30 min at room temperature with dithiobis(succinimidylpropionate) (DSP) prior to rupture and immunoadsorption of heterocomplexes. Cells treated with the DSP crosslinker yielded 2.5 times the amount of nNOS-FLAG•Hsp90 heterocomplexes as cells that were not treated with DSP. Thus, without the crosslinker, about two-thirds of the apo-nNOS•Hsp90 heterocomplexes had dissociated in untreated cells. Although this clearly shows the dynamic nature of the complex, to avoid complications associated with crosslinking, all subsequent experiments were performed without crosslinking.

Figure 2*B* shows that incubation of stripped apo-nNOS-FLAG immunopellets with Sf9 cytosol yields heterocomplex assembly with Hsp90 and Hsp70 and that heterocomplex assembly is better when an ATP-regenerating system (ARS) is supplemented to the cytosol.

In the experiments charted in Figure 2*C*, immunoadsorbed and purified apo-nNOS-FLAG was incubated first with Sf9 cytosol, to reconstitute the chaperones onto the immobilized client protein, the immunopellets were washed, and incubated a second time with the heme-containing nNOS activation system. nNOS activity was then assayed on the material released by incubation with FLAG peptide. Apo-nNOS-FLAG activation is somewhat better when ARS is supplemented to the cytosol (*cf*., *lanes* 3 and 4), and there is no activation when heme is not present in the second incubation with the activation system (*lane* 5). The level of activation achieved is about one-third of that of holo-nNOS (*cf*., *lanes* 3 and 6). Our conclusion from Figure 2, *B* and *C* is that Sf9 cytosol can both form apo-nNOS-FLAG•Hsp90•Hsp70 heterocomplexes and activate the enzyme.

Figure 2*D* shows that we can improve on the extent of activation with alteration of the experimental protocol. In this case, the apo-nNOS-FLAG immunopellets were incubated with both Sf9 cytosol and the heme-binding nNOS activation system together in the same incubation mix. Our objective was to have heme enter as the apo-nNOS-FLAG was undergoing dynamic assembly/disassembly with Hsp90 and thus trap evidence of heme insertion before dissociation of the apo-enzyme–Hsp90 heterocomplex occurred. As Figure 2*D* shows, this protocol allows enzyme activation to nearly the level of purified holo-nNOS-FLAG.

To obtain evidence that Hsp90 and Hsp70 are critical for nNOS activation, enzyme activation under the protocol of Figure 2*D* was carried out in the presence of radicicol, a specific inhibitor of Hsp90 (43), or pifithrin-μ, a specific inhibitor of Hsp70 (44). As shown in Figure 2*E*, each inhibitor reduced apo-nNOS-FLAG activation by about 60% and the combination by 85% without affecting the activity of purified holo-nNOS-FLAG. As shown in Figure 2*F*, to further verify the role of Hsp90, we added back purified Hsp90 to the radicicol-treated sample. The inhibition caused by radicicol was nearly fully reversed by addition of purified Hsp90 (*solid circles*), whereas there was no effect of Hsp90 alone (*open circles*). Moreover, treatment with radicicol leads to a loss of Hsp90, but not Hsp70, bound to nNOS (Fig. 2*F*, *inset*). Thus, it appears that the fresh Hsp90 must serve to replace the Hsp90 that was removed by radicicol. These studies further support the specificity of radicicol and the role of Hsp90 in heme-mediated activation. Although Hsp70 has been shown to be involved in the *in vitro* heme-mediated activation of purified apo-nNOS (27), this is the first time we have been able to show a role for Hsp90 in an *in vitro* system.

### Reconstitution of nNOS heterocomplexes with the purified chaperone machinery

Although the Sf9 cytosol allows cycling of Hsp90 and Hsp70 with apo-nNOS-FLAG to demonstrate both heterocomplex assembly with the chaperones and chaperone-dependent and heme-mediated activation of enzyme activity, the Sf9 system does not facilitate identification of the machinery required for dynamic assembly of the apo-nNOS-FLAG•chaperone complex. For this purpose, we want to form heterocomplexes and promote heme-dependent activation with purified chaperone proteins. During the 1990s, the components for generating the steroid-binding activity of GRs and progesterone receptors were isolated and reconstituted to yield an efficient Hsp90 heterocomplex assembly system of five proteins—Hsp90, Hsp70, Hop, Hsp40, and p23 (1). This has proven to be a facile system for defining the role of proteins in the chaperone machinery (45).

**Figure 3:**
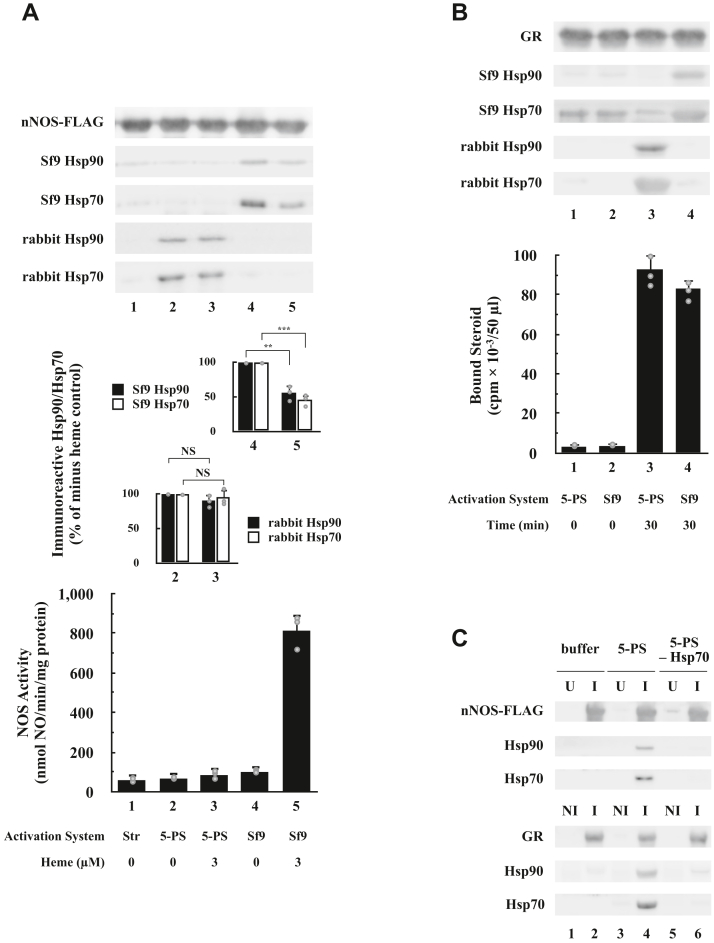
**Assembly of apo-nNOS•Hsp90•Hsp70 heterocomplexes with the 5-protein system (5-PS).***A*, comparison of the 5-PS and Sf9 cytosol at nNOS-FLAG heterocomplex assembly and heme-dependent nNOS enzyme activation. Purified apo-nNOS-FLAG was immunoadsorbed with anti-FLAG and stripped of associated chaperones by incubating for 5 min at 30 °C with the ATP-regenerating system. *Lane* 1 consists of a stripped (*Str*) immunopellet in buffer alone. *Lanes* 2 and 3, stripped immunopellets incubated with the *5*-PS and the activating mixture without (*lane* 2) or with (*lane* 3) 3 μM heme. *Lanes* 4 and 5, stripped immunopellets incubated with Sf9 cytosol without (*lane* 4) or with (*lane* 5) heme. Apo-nNOS-FLAG and associated chaperones were released with FLAG peptide and resolved by SDS-PAGE and immunoblotting with a sample Western blot shown at the *top* of *A*. Hsp90 and Hsp70 bands from three gels were scanned, and the amount of Hsp90 (*solid bars*) or Hsp70 (*open bars*) in the heme-containing samples was expressed as a percentage of the non–heme-containing samples for both the Sf9 cytosol samples (*top inset bar graph*) and for the 5-PS (rabbit Hsp90 and Hsp70, *bottom inset bar graph*). NS, not significant; ∗∗, difference significant at *p* < 0.01; ∗∗∗, difference significant at *p* < 0.001. nNOS activity was assayed for all conditions as shown in the *large bar graph at the bottom*. The nNOS activities represent the mean ± SD for three experiments. *B*, comparison of Sf9 cytosol and the 5-PS for GR heterocomplex assembly and activation of steroid-binding activity. GR was immunoadsorbed from replicate aliquots of GR-expressing Sf9 cytosol, stripped of associated chaperones, and immunopellets were incubated for 0 (*lanes* 1 and 2) or 30 min (*lanes* 3 and 4) at 20 °C with the 5-PS or with Sf9 cytosol (*Sf9*). GR•chaperone heterocomplexes were measured by immunoblotting (*top*), and steroid binding was assayed (*bar graph*) (mean ± SD for three experiments). *C*, Hsp70 is required for assembly of nNOS or GR chaperone heterocomplexes by the 5-PS. The assembly and blotting were as described above. For nNOS experiments (*upper panel*), untagged apo-nNOS (U) was used for controls (*lanes* 1, 3, and 5). For GR experiments (*lower panel*), nonimmune antibody (NI) was used for controls (*lanes* 1, 3, and 5). *Lanes* 1 and 2 consist of a stripped immunopellet incubated with buffer alone. *Lanes* 3 and 4, stripped immunopellets incubated with the 5-PS. *Lanes* 5 and 6, stripped immunopellets incubated with Hsp70 omitted from the 5-PS *(5PS-Hsp70*). GR, glucocorticoid receptor; Hsp, heat shock protein; nNOS, neuronal nitric oxide synthase.

Figure 3*A* compares assembly of nNOS-FLAG•chaperone heterocomplexes and enzyme activation by the 5-protein system and Sf9 cytosol. As shown by the immunoblot at the *top* of Figure 3*A*, the 5-protein system assembles nNOS-FLAG heterocomplexes both in the absence (*lane* 2) and in the presence (*lane* 3) of the heme-mediated activating system. But, as shown by the *solid bars* at the very *bottom* of Figure 3*A*, no enzyme activation occurs (*lane* 3). In contrast, Sf9 cytosol assembles nNOS-FLAG heterocomplexes with Hsp90 and Hsp70 in the absence (*lane* 4) and presence (*lane* 5) of heme, and when heme is present, enzyme activation occurs (*lane* 5 in the *bar graph* at the *bottom*). Consistent with observations made on other heme proteins (30, 32, 34), much less Hsp90 was recovered with the enzymatically active holo-nNOS produced by the Sf9 cytosol (*solid bar* in *lane* 5 of the *inset* in the *middle* of Fig. 3*A*) than was recovered with the apo-nNOS (*solid bar*, *lane* 4 in the *inset*). As indicated by the *open bars* in the same *inset*, less Hsp70 is recovered with the holo-nNOS as well. In contrast, similar amounts of Hsp90 and Hsp70 are recovered in heterocomplexes assembled by the 5-protein system in the presence and absence of heme (*cf*., *lane* 3 with *lane* 2 in the *lower bar graph* of the *inset*).

At this point, it was important to determine that the 5-protein system we are using was active in promoting ligand binding by a non–heme-binding Hsp90 client protein. Figure 3*B* compares the activity of the 5-protein system with Sf9 cytosol in both GR heterocomplex assembly with Hsp90 and Hsp70 and activation of GR ligand-binding activity. As shown in the Western blot at the top of the figure, both the 5-protein system (*lane* 3) and Sf9 cytosol (*lane* 4) formed GR•Hsp90•Hsp70 complexes. Also, as illustrated in the bar graph at the *bottom*, both systems activated steroid-binding activity.

To further verify the role of Hsp70 in the assembly of the apo-nNOS chaperone heterocomplex, we utilized the 5-protein system. As shown in Figure 3*C* (*upper panel*), the assembly of Hsp90 onto the apo-nNOS was entirely dependent on Hsp70 (*cf*., *lane* 4 with *lane* 6). This is entirely consistent with the K^+^ dependency of the assembly process seen earlier with dialyzed reticulocyte lysate. As a control (*lower panel*), we compared the GR under highly similar conditions to reveal the same Hsp70 dependence (*cf*., *lane* 4 with *lane* 6). Thus, the nNOS and GR share a similar chaperone heterocomplex.

**Figure 4:**
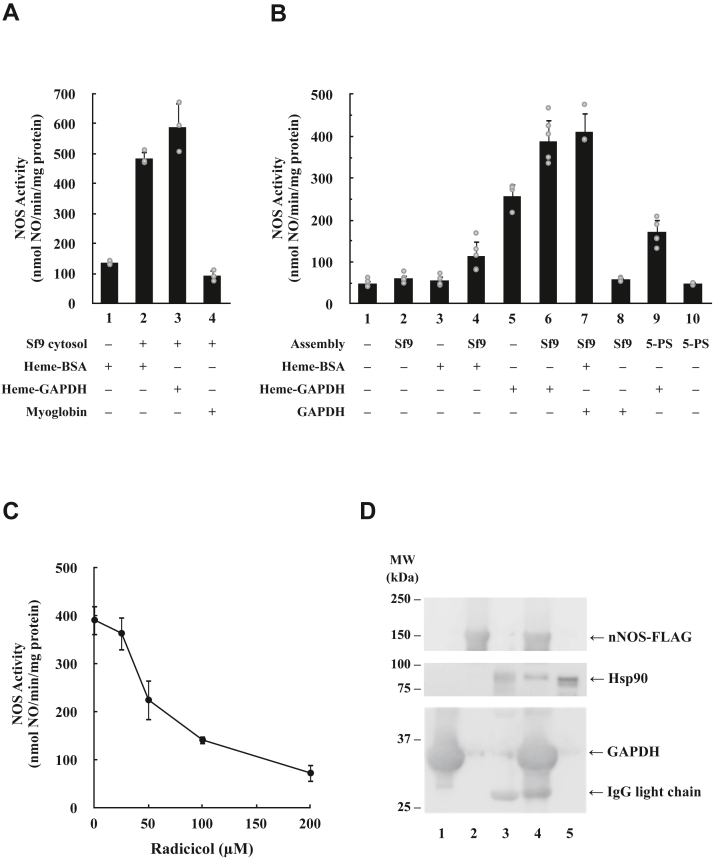
**Comparison of heme-GAPDH, heme-BSA, and myoglobin as heme donors in the activation of apo-nNOS-FLAG with Sf9 cytosol or the Sf9 reconstitution system.***A*, heme-GAPDH and heme-BSA, but not myoglobin, act as heme donors in the activation of apo-nNOS-FLAG by Sf9 cytosol. The conditions are as for Figure 2*D* except that Sf9 cytosol was at 0.75 mg/ml, and heme-GAPDH or myoglobin was substituted for heme-BSA as indicated. The final heme concentrations in each case were 3 μM. The nNOS activities represent the mean ± SD for three experiments. *B*, heme-GAPDH, but not heme-BSA, acts as a heme donor in the activation catalyzed by the Sf9 reconstitution system. The experiments were performed as for Figure 2*C*. Immunoadsorbed purified apo-nNOS-FLAG was incubated with ARS and either buffer alone (*lanes* 1, 3, and 5), or with Sf9 cytosol to assemble the chaperone complex (*lanes* 2, 4, 6, 7, and 8), or with the 5-protein system to assemble a purified protein complex (*lanes* 9 and 10). The immunopellet was washed and then incubated with buffer (*lanes* 1, 2, and 10) or heme-BSA (*lanes* 3 and 4), heme-GAPDH (*lanes* 5, 6, and 9), heme-BSA and GAPDH (*lane* 7), or GAPDH (*lane* 8). The nNOS activities represent the mean ± SD for at least three experiments. *C*, radicicol inhibits the heme-GAPDH–mediated activation of apo-nNOS-FLAG by the Sf9 reconstitution system. The experiment was as described for *B* (*lane* 6), except that the indicated amounts of radicicol were added at the same time as heme-GAPDH. The nNOS activities represent the mean ± SD for three experiments. *D*, the presence of Hsp90 in anti-FLAG M2 affinity gel. About 20 μg of purified GAPDH (*lane* 1), 2.5 μg of purified apo-nNOS-FLAG (*lane* 2), 5 μl of M2 affinity gel (*lane* 3), the entire reaction mixture from the sample in lane 5 from *B* (*lane* 4), and 0.2 μg of purified Hsp90 (*lane* 5) were immunoblotted for nNOS, GAPDH, and Hsp90. ARS, ATP-regenerating system; BSA, bovine serum albumin; Hsp, heat shock protein; nNOS, neuronal nitric oxide synthase.

We conclude from Figure 3 that the 5-protein system is capable of nNOS heterocomplex cycling with Hsp90 but lacks a factor (or factors) present in Sf9 cytosol that promotes functional heme insertion to form holo-nNOS and enzyme activation. Studies in cells showing GAPDH acts to donate heme to iNOS, soluble guanylate cyclase, and hemoglobin (36, 37, 38, 39, 46) prompted us to test if this protein was one of the missing factors. As shown in Figure 4*A*, heme–GAPDH can substitute for heme-BSA as the heme donor in the heme-mediated activation of apo-nNOS that occurs in the presence of Sf9 cytosol (*cf*., *lane* 2 with *lane* 3). Clearly, myoglobin, which tightly holds on to its heme prosthetic group, does not substitute as a heme donor, and we find no activation (*cf*., *lane* 4 with *lane* 1). Interestingly, when we tested for the ability of heme-GAPDH to activate the immunoadsorbed apo-nNOS-FLAG•chaperone heterocomplexes formed from Sf9 reconstitution system previously described in Figure 2*C*, we found that heme-GAPDH was far better at activation than heme-BSA (Fig. 4*B*, *cf*., *lane* 6 with *lane* 4). Moreover, addition of the heme-GAPDH activated apo-nNOS by itself, although less well than when the apo-nNOS-FLAG•chaperone heterocomplexes was present (*cf*., *lane* 5 with *lane* 6). This is consistent with the notion that GAPDH acts as a heme chaperone. Since GAPDH itself had a substantial effect (*lane* 5), we wondered about the role of the Hsp90-based chaperone system on the overall heme-mediated activation. As shown in Figure 4*C*, radicicol inhibited the action of heme-GAPDH on the immunoadsorbed nNOS-FLAG•chaperone heterocomplexes formed from Sf9 reconstitution system, to nearly that of the no heme control. Thus, we not only confirm that Hsp90 is still required in the activation of immunoadsorbed nNOS-FLAG–chaperone heterocomplexes by heme-GAPDH, but the nearly complete inhibition by radicicol suggests that we have contaminating Hsp90 in the immunopellet in the sample in Figure 4*B* (*lane* 5). As shown in Figure 4*D*, we verified by Western blotting that this immunopellet has Hsp90 (*lane* 4), and furthermore, the contaminating Hsp90 is not from GAPDH (*lane* 1) or nNOS-FLAG (*lane* 2) but from the anti-FLAG M2 agarose preparation (*lane* 3). Thus, Hsp90 is required for heme-mediated activation of apo-nNOS even with the heme chaperone GAPDH.

We suspect that the activation seen with heme-BSA in the previous experiment in the presence of cytosol is due to the transfer of heme to GAPDH, which is present in large amounts in Sf9 cytosol. The ability for heme-BSA to activate the apo-nNOS heterocomplex in the presence of Sf9 cytosol was restored when we added heme-free GAPDH (Fig. 4*B*, *cf*., *lane* 7 to *lane* 4), thus showing that this transfer is possible. As expected, the control of adding the heme-free GAPDH alone did not have an effect on the apo-nNOS heterocomplex (*lane* 8). Thus, we conclude that it is GAPDH that is mainly responsible for the activation of apo-nNOS under all conditions examined.

Unfortunately, the apo-nNOS complex with the purified 5-protein system is still not sufficient to activate apo-nNOS even with heme-GAPDH, and in fact seemed to be slightly inhibitory (*cf*., *lane* 9 to *lane* 5). Thus, we are still unable to fully reconstitute the heme insertase chaperone machinery with purified proteins. Nonetheless, our studies show that heme-GAPDH is the obligatory heme donor and needs to work with Hsp90•Hsp70 chaperones to carry out the heme insertion and activation of apo-nNOS.

## Discussion

Studies in cells have established a role for Hsp90 in the insertion of heme into apo-nNOS to form the active holo-nNOS enzyme (29). Because of the highly dynamic nature, neither we have been able to study the interaction of Hsp90 with apo-nNOS nor we have been able to demonstrate the Hsp90-dependent heme insertion process with the use of only subcellular systems (27). In the current study, we discovered that Hsp90 and Hsp70 heterocomplexes could be assembled onto an antibody-immobilized apo-nNOS client protein with the use of subcellular fractions from reticulocytes and Sf9 cells as well as with purified proteins. Furthermore, the Hsp90-dependent heme insertion and activation of apo-nNOS could be demonstrated for the first time in Sf9 cytosol when the immobilized apo-nNOS was used. The exact reason that immobilized client proteins are needed is not known; however, this is the method used in the study of the Hsp90 and Hsp70 chaperones and the steroid receptors (35).

The kinetics of assembly onto immobilized apo-nNOS by reticulocyte lysate revealed the rapid initial binding of Hsp70 followed by the slower binding of Hsp90 by a process that required both ATP and K^+^. As elucidated for the GR, the K^+^ is required for Hsp70 to cycle and load Hsp90 (41) and is consistent with the need for Hsp70 to load the Hsp90 onto apo-nNOS. In addition, when we substituted Sf9 cytosol for purified Hsp90, Hsp70, Hsp40, Hop, and p23, we were able to assemble the Hsp90 and Hsp70 onto the apo-nNOS, similar to that found for the GR (1, 45, 47). Consistent with the K^+^ dependence, Hsp70 was needed for assembly of nNOS–Hsp90 heterocomplexes by this purified protein system highly similar to that found for GR. Thus, there are many similarities with that found for GR•Hsp90•Hsp70 heterocomplex assembly process, and it is highly likely that apo-nNOS interacts with the same chaperone machinery. However, as will be discussed later with the use of purified chaperones, there is a fundamental difference when it comes to the Hsp90-dependent chaperone machinery needed for steroid insertion into the steroid receptor to the Hsp90-dependent chaperone machinery needed for heme insertion.

The immobilized apo-nNOS was efficiently activated when heme-BSA was added in the presence of Sf9 cytosol. This process was inhibited by specific inhibitors to Hsp90 and Hsp70 chaperones, albeit at relatively higher concentrations than normally used in studies on the steroid receptor. Higher concentration of Hsp90 inhibitor, radicicol, was needed in previous studies with the use of intact cells expressing apo-nNOS as well (26). This was attributed to the dynamic nature of the Hsp90 interaction with nNOS. Of course, at higher concentrations, radicicol may have nonspecific effects; so we rescued from radicicol-mediated inhibition with the addition of purified Hsp90. Thus, we concluded that heme-mediated activation was Hsp90 dependent in Sf9 cytosol and recapitulated the cellular process.

Unlike that found with crude cytosol, the purified protein system comprising of Hsp90, Hsp70, Hsp40, Hop, and p23 did not facilitate the heme-mediated activation of apo-nNOS, even though we showed that this system could insert steroid into the GR. This represents a fundamental difference between heme and steroid insertion into their respective client proteins. We do not yet know if we are missing a critical factor that is needed in addition to the five purified proteins or if one or more of the five purified proteins negatively affects heme insertion and activation of apo-nNOS.

In the course of our studies to find a missing factor, we examined GAPDH as it was shown to be a heme-binding protein and acts as a heme chaperone in cells (33, 36, 37, 38, 39). In all our previous studies, we have used heme-BSA as the heme donor and failed to see a difference between heme-GAPDH and heme-BSA. Interestingly, in the current study, the immobilized apo-nNOS-FLAG•Hsp90•Hsp70 chaperone heterocomplex formed from the Sf9 reconstitution system could catalyze the heme-mediated activation of apo-nNOS only when heme-GAPDH, but not heme-BSA, was present. Thus, there was a clear preference for heme-GAPDH in biochemical assays that likely reflects the cellular process.

Although the anti-FLAG M2 affinity gel had contaminating Hsp90 that limited the experiments, we determined with the use of radicicol that Hsp90 is still required for the heme-GAPDH to activate apo-nNOS. Taken together, GAPDH is the obligate heme donor that must work together with Hsp90-based chaperone complex to insert heme and activate apo-nNOS. The mechanism of how GAPDH and Hsp90 cooperate to elicit this function can now be biochemically dissected with the use of subcellular fractions. This is important as studies with cells by Stuehr laboratory have shown that Hsp90 and GAPDH are involved in determining the heme content and thus function of a variety of other critical hemeproteins (30, 31, 32, 33, 34, 36, 37, 38, 39). Interestingly, a recent study found that heme insertion into tryptophan dioxygenase is GAPDH dependent but Hsp90 independent (33). Perhaps the model system developed here may be useful in elucidating how GAPDH and Hsp90-based chaperones work together in some cases and not in other cases. In that the highly transient interactions captured in our studies are thought to be a common mode of interaction for Hsp90-based chaperones (2, 20), our studies may provide insight into how Hsp90 regulates many client hemeproteins. Future work is still needed to fully replicate the heme insertase machinery with purified proteins.

## Experimental procedures

### Materials

Anti-FLAG M2 affinity gel, high-activity potato apyrase, radicicol, and rabbit GAPDH were purchased from Sigma–Aldrich. FLAG octapeptide (N-Asp-Tyr-Lys-Asp-Asp-Asp-Asp-Lys-C) was synthesized by the Proteomics & Peptide Synthesis Core at The University of Michigan. Purified mouse monoclonal anti-nNOS immunoglobulin G (IgG)_2a_ used to immunoblot nNOS was from BD Biosciences. Rabbit polyclonal anti-Sf9 Hsp90 antiserum and purified rabbit polyclonal anti-Sf9 Hsc70/Hsp70 IgG were purchased from Lampire Biological Laboratories. Mouse anti-Hsp90 monoclonal antibody (AC88) and mouse anti-Hsc70/Hsp70 monoclonal antibody (N27F3-4) used for immunoblotting the rabbit and human proteins were from Enzo Life Sciences. The ascites containing mouse monoclonal IgG (FiGR) used to immunoadsorb the mouse GR were produced at University of Michigan Hybridoma Core with FiGR hybridoma cells obtained from American Type Culture Collection. The BuGR2 monoclonal IgG used to immunoblot the mouse GR and DSP were purchased from Thermo Fisher Scientific. Mouse monoclonal anti-GAPDH antibody (D-6) was from Santa Cruz Biotechnology. IRDye 800CW-conjugated goat polyclonal antimouse IgG and IRDye 680LT-conjugated goat polyclonal anti-rabbit IgG used for quantitating immunoblot bands were purchased from LI-COR. [1,2,4,6,7-^3^H] Dexamethasone (77 Ci/mmol) was from PerkinElmer.

### Methods

#### Cell culture and cytosol preparation

Sf9 cells were grown in Sf-900 II SFM serum-free, protein-free insect cell culture medium (Thermo Fisher Scientific) supplemented with 1% (v/v) bovine calf serum in suspension cultures maintained at 27 °C with continuous shaking (160 rpm). The Sf9 cells in growing phase were infected for 72 h with 0.2% (v/v) of baculovirus containing complementary DNA encoding rat nNOS that is tagged at C terminus with FLAG peptide as previously described (48), being referred to as nNOS-FLAG. For production of holo-nNOS-FLAG, hemin (7 μM) and *N*-acetyl-l-cysteine (2 mM) were added 6 h prior to harvesting as an albumin conjugate, prepared as described previously (7). Harvested cells were washed and pelleted (900*g*), and the volume was measured. The cells were sonicated on ice in 1.5 times the pellet volume of HEM buffer (10 mM Hepes, pH 7.35, 1 mM EDTA, 20 mM sodium molybdate) containing one tablet/10 ml cOmplete Mini protease inhibitor cocktail (Roche Applied Science) and 1 mM PMSF in the presence or the absence of 10 μM tetrahydrobiopterin for holo-nNOS-FLAG or apo-nNOS-FLAG, respectively. The resultant whole-cell lysate was ultracentrifuged at 4 °C for 20 min at 200,000*g*. The supernatant, being referred to as cytosol, was collected, aliquoted, and flash-frozen at −80 °C.

#### Immunoadsorption of nNOS-FLAG and nNOS•Hsp90•Hsp70 heterocomplex assembly

Anti-FLAG M2-agarose gel was equilibrated with TEGM buffer (10 mM TES, pH 7.6, 50 mM NaCl, 1 mM EDTA, 10% [v/v] glycerol, 20 mM sodium molybdate). Cytosol of uninfected or nNOS-expressing Sf9 cells was incubated by rotation with M2-agarose gel for 90 min at 4 °C. The agarose gel was washed twice with TEGM buffer and once with Hepes buffer (10 mM Hepes, pH 7.35). Immunoadsorbed nNOS-FLAG was stripped of endogenous Sf9 Hsp90 and Hsp70 by incubation for 5 min at 30 °C with an ARS (10 mM Hepes, pH 7.4, 50 mM ATP, 250 mM creatine phosphate, 20 mM magnesium acetate, and 100 units/ml creatine phosphokinase) as described previously (47). The agarose gel was washed twice with TEG buffer (10 mM TES, pH 7.6, 50 mM NaCl, 1 mM EDTA, 10% [v/v] glycerol) and once with Hepes buffer. To form nNOS•Hsp90•Hsp70 heterocomplexes, stripped nNOS-FLAG was incubated for 20 min at 30 °C in the presence of an ARS with cytosol of uninfected Sf9 cells prepared in HKD buffer (10 mM Hepes, pH 7.35, 100 mM KCl, and 0.1 mM DTT) or rabbit reticulocyte lysate with frequent agitation. The agarose gel was then washed four times with TEGM buffer for immunoblotting or twice for subsequent heme-mediated activation. The FLAG-tagged nNOS•Hsp90•Hsp70 heterocomplexes were eluted from the M2 beads by incubation with 150 μM FLAG peptide for 15 min at 4 °C in HEM buffer. The eluted samples were treated with Laemmli sample buffer containing 10% (v/v) 2-mercaptoethanol and submitted to SDS-PAGE and immunoblotting.

#### Treatment of rabbit reticulocyte lysate

Reticulocyte lysate was dialyzed (molecular weight cutoff: 10 kDa) overnight against Hepes buffer containing 1 mM DTT followed by a second 2 h dialysis against fresh buffer. Dialyzed lysate was concentrated to one-half the original volume by Amicon Ultra centrifugal concentrators (molecular weight cutoff: 30 kDa). In some experiments, the endogenous ATP in cytosol or lysate was eliminated by treatment for 30 min at 30 °C with 0.2 unit/100 μl of apyrase as described previously (41).

#### Assay of catalytic activity of reconstituted nNOS•Hsp90•Hsp70 heterocomplexes

Following the reconstitution reaction, nNOS•Hsp90•Hsp70 heterocomplexes assembled on M2-agarose gel were washed twice with TEGM buffer and incubated for 90 min at room temperature with a heme-containing nNOS activation system consisting of 10 mM Hepes, pH 7.35, 100 mM KCl, 0.1 mM DTT, 0.1 mM EGTA, 50 μM tetrahydrobiopterin, 20 units/ml superoxide dismutase, 200 units/ml catalase, 1 μM heme as a hemin–BSA complex, and 1 μM Hip. Where indicated, heme-GAPDH was prepared according to previously described methods (46) and substituted for heme-BSA. Immunopellets were washed twice with TEGM buffer containing 0.1 mM DTT and 10 μM tetrahydrobiopterin, and nNOS•Hsp90•Hsp70 heterocomplexes were eluted from the M2-agarose beads as described previously. NO formation was assayed by the NO-mediated conversion of oxyhemoglobin to methemoglobin as described previously (27). Briefly, 20 μl of the eluted nNOS•Hsp90•Hsp70 heterocomplexes were added to 180 μl of 50 mM Tris, pH 7.4, 200 μM CaCl_2_, 250 μM l-arginine, 100 μM tetrahydrobiopterin, 100 units/ml catalase, 5 μg/ml CaM, 25 μM oxyhemoglobin, and an NADPH-regenerating system consisting of 400 μM NADP^+^, 10 mM glucose-6-phosphate, and 1 unit/ml glucose-6-phosphate dehydrogenase. The mixture was incubated at 37 °C, and the rate of oxidation of oxyhemoglobin was monitored by measuring the absorbance at λ_401−411 nm_ with a microtiter plate reader. nNOS activity is expressed as nanomole NO/min/mg protein ± SD for three or more experiments.

#### Gel electrophoresis and Western blotting

Immunopellets were resolved on 12% SDS-polyacrylamide gels and transferred to Immobilon-FL membranes. The membranes were probed with 1.0 μg/ml BuGR2 for GR, 0.1% (v/v) anti-Sf9 Hsp90 antiserum for Sf9 Hsp90, 1.0 μg/ml anti-Sf9 Hsp70 IgG, 2.5 μg/ml AC88 for rabbit Hsp90, 1.0 μg/ml N27F3-4 for rabbit Hsp70, and 1.0 μg/ml D-6 for GAPDH for 1 h at room temperature. The immunoblots were then incubated a second time with IRDye 800CW-conjugated goat antimouse IgG (H + L) for GR, rabbit Hsp90, and rabbit Hsp70, and IRDye 680LT-conjugated goat anti-rabbit IgG (H + L) for Sf9 Hsp90 and Hsp70. Washed membranes were scanned by Odyssey Fc Imaging System (LI-COR), and the immunoreactivity was quantitated by Image Studio (LI-COR) software within a linear dynamic range for each protein.

#### Protein purification and incubation with the 5-protein system

Hsp70, Hop, YDJ-1 (the yeast ortholog of Hsp40), and p23 were purified as described (49). The mixture of rabbit Hsp90α and Hsp90β was purified from rabbit reticulocyte lysate as described (49). GRs were immunoadsorbed from aliquots of 50 μl (for measuring steroid binding) or 100 μl (for Western blotting) of Sf9 cell cytosol by rotation for 2 h at 4 °C with 14 μl of protein A-Sepharose precoupled with 2 μl of FiGR antibody suspended in 200 μl of TEG buffer. Immunoadsorbed GR was stripped of endogenously associated Hsp90 by incubating the immunopellet for an additional 2 h at 4 °C with 350 μl of 0.5 M NaCl in TEG buffer. The pellets were then washed once with 1 ml of TEG buffer followed by a second wash with 1 ml of Hepes buffer. For heterocomplex reconstitution with purified proteins, immunopellets containing GR or apo-nNOS stripped of chaperones were incubated with 20 μg of purified rabbit Hsp90, 15 μg of purified rabbit Hsp70, 0.6 μg of purified human Hop, 0.125 μg of purified yeast YDJ-1, and 6 μg of purified human p23 adjusted to 55 μl with HKD buffer containing 20 mM sodium molybdate and 5 μl of an ARS. The assay mixtures were incubated for 20 min at 30 °C with suspension of the pellets by shaking the tubes every 2 min. At the end of the incubation, the pellets were washed twice with 1 ml of ice-cold TEGM buffer and assayed for steroid-binding capacity and for GR-associated or apo-nNOS-associated Hsp90 and Hsp70.

#### Assay of steroid-binding capacity

Washed immunopellets to be assayed for steroid binding to stable GR•Hsp90 heterocomplexes were incubated overnight at 4 °C in 50 μl of HEM buffer plus 50 nM [^3^H]dexamethasone. Samples were then washed three times with 1 ml of TEGM buffer and counted by liquid scintillation spectrometry. The steroid binding is expressed as counts/min of [^3^H]dexamethasone bound/50 μl of cytosol.

## Data availability

All data are contained within the article.

## Conflict of interest

The authors declare that they have no conflicts of interest with the contents of this article.
